# Feeling Rested Improves Cognitive Performance Among University Students: Testing of a Novel Psychophysiological Measurement System

**DOI:** 10.3390/brainsci16020136

**Published:** 2026-01-27

**Authors:** Márk Komóczi, Levente Lévai, Péter Barna, Karolina Kósa

**Affiliations:** 1 Department of Behavioural Sciences, Faculty of Medicine, University of Debrecen, Móricz Zsigmond krt. 22, 4032 Debrecen, Hungary; 2 Aviatronic Ltd., Konkoly-Thege út 29-33, 1121 Budapest, Hungary

**Keywords:** cognitive tests, gamified psychological test, heart rate variability, psychophysiology, sleep duration

## Abstract

Background: Academic performance is related to cognitive functions and satisfied physiological needs such as proper sleep, a factor frequently overlooked by university students. Our aim was to investigate sleep-related variables, cognitive performance and stress level measured by heart rate variability among university students. Methods: A novel psychophysiological measurement system was used for data collection in which a screen-adapted questionnaire was used to collect data on sleep; gamified versions of standard psychological tests were used to assess cognitive performance, and ECG data were recorded by a wearable ECG sensor, all synchronized by a software. University students volunteered for anonymous testing that lasted approximately one hour. Results: Of the 107 students (mean age: 22.2 years, *SD* ± 2.22; 52% female), those who reported being well-rested achieved significantly higher overall cognitive performance (*p* = 0.024). Sleep duration did not correlate with cognitive performance but longer sleep duration was associated with feeling rested (*rho* = 0.326; *p* < 0.001). Cognitive performance showed significant association with two HRV parameters such as the Baevsky Stress Index (*r* = 0.195), higher values of which reflect higher autonomic stress load. Significant negative relation was found between cognitive performance and RMSSD (*r* = −0.195), another HRV parameter, higher values of which allude to higher parasympathetic activity (*p* = 0.050 for both). These findings suggest a link between mild arousal and performance. Conclusions: Being rested and lower autonomic stress load are positively correlated with cognitive performance. The novel psychophysiological measurement system integrating subjective and objective measurements of cognitive and physiological functions is feasible for assessing cognitive functions and stress levels in students.

## 1. Introduction

### 1.1. Cognitive Functions

Cognitive functions are involved in knowledge acquisition by decoding information through perception, attention, reasoning, memory, learning, decision-making and language. Tests assessing cognitive functions have been used in various fields such as patient care, occupational health, and choice of career [1,2]. Gamification of these tests for computer screens increases participant motivation and engagement [3]. Since acute and chronic stress tend to negatively impact cognitive performance, the synchronous measurement of cognitive performance and stress would be of great importance.

### 1.2. Cardiac Activity as Indicator of the State of the Autonomic Nervous System

Several research groups from the 1970s onward began exploring the possibilities of the quantitative characterization of heart rate variability (HRV), the normal variation in beat-to-beat duration of the R-R interval associated with breathing also known as physiological respiratory sinus arrhythmia [4,5] as an indicator for stress assessment. Though there is no universally accepted standard method for measuring stress, current scientific evidence supports the use of HRV as an objective parameter for stress assessment [6]. Guidelines for the measurement, evaluation, and clinical application of HRV were released as early as 1996 by the European and North American cardiology societies [7]. Since then, other research [8] and clinical [9] guidelines about the use of HRV have been published.

As an increasing number of wearable sensors—among the ECG sensors [10,11]—became available for general and healthcare use from the beginning of the 21st century, it became possible to monitor cardiac activity including changes in HRV in real time in various situations [12].

### 1.3. The Relationship Between Cognitive and Physiological Functions

Investigation of the relationship between cognitive functions and the autonomic nervous system began just over a decade ago. In 2009, Thayer and colleagues published their model of neurovisceral integration, according to which HRV is a particularly sensitive indicator of the flexible neural prefrontal network that plays a key role in adapting to different situations [13].

A systematic review covering 20 studies on the relationship between HRV and cognitive functions found that declines in cognitive functions are associated with increased sympathetic and decreased parasympathetic activity even in the absence of dementia, severe cardiovascular disease, or other major illnesses [14]. However, the relationship between certain specific cognitive functions, such as executive functions and the autonomic nervous system remains under investigation in terms of whether autonomic dysfunction is a cause or a consequence of cognitive impairment [14,15]. This dilemma can only be settled by analytical (as opposed to descriptive) studies.

According to current knowledge, heart rate variability (HRV) is not only a cardiovascular marker of current stress but is also associated with self-reported stress [16]. A literature review from 2023 on the relationship between HRV, neurological status and cognition concluded that the heart and brain have a bidirectional relationship. Balanced interaction indicates good health, whereas regulatory problems—such as reduced vagal function or decreased heart rate variability—may be associated with cognitive dysfunction or other neurological impairments [17].

Patients with psychiatric disorders exhibit complex cognitive deficits which negatively affect daily functioning and tend to worsen with advancing age [18]. Individuals with psychiatric disorders exhibit a significant decline in HRV with age; however, it is unclear whether this decrease is a consequence of aging or of the disorder itself [19].

### 1.4. Lack of Sleep and Cognitive Performance in University Students

Sleep as a biological function has been shown to be generally related to health and wellbeing [20], particularly memory formation [21], decision-making [22] and work capacity [23] though its neurobiological foundations and processes are still being researched [24]. Despite the scientific understanding of its importance, sleep deprivation seems to be a global problem affecting one-third of adults in the US [25]. University students also often experience disturbed sleep patterns that are related to high stress levels and mental health challenges [26,27]. This is particularly concerning in this group because experimental studies revealed that sleep deprivation significantly impairs cognitive performance in college students, particularly on tasks involving working memory and executive functioning [28]. Longitudinal research demonstrated that poorer sleep quality and greater psychological distress are associated with decrements in working memory, despite stable or even elevated academic performance, suggesting varying levels of cognitive resilience among students [29]. All these findings underscore the intricate interplay among sleep, mental health and cognition in university students for whom optimal cognitive performance is a major determinant of their future life.

### 1.5. Objectives

To analyze the relationship between cognitive performance as an objectively assessed major outcome and the feeling of being rested as its determinant, we assessed sleep duration and subjective perception of being rested along with objective measurements of heart rate variability in university students hypothesizing that both sleep duration and feeling rested would be associated with better cognitive performance.

## 2. Materials and Methods

### 2.1. Study Design

A cross-sectional study was designed as part of an ongoing research project to investigate cognitive performance and psychophysiological functioning by using a novel psychophysiological test system. The current analysis focused on sleep-related variables, cognitive performance and heart rate variability. Ethics approval was issued by the National Center for Public Health and Pharmacy (NNGYK/GYSZ/34244-2/2024).

### 2.2. Participants

Full-time active students of bachelor, master, doctoral levels not on sick leave, studying in Hungarian at any of the 13 faculties of the University of Debrecen, were invited to participate in order to create a relatively homogeneous sample. Expecting an effect size of 0.3 (correlation coefficient for the hypothesized association), a sample of 85 persons was calculated [30].

### 2.3. Measurement System and Data Collection

The non-invasive measurement system consisted of various questionnaires and standard gamified cognitive tests adapted to a computer screen that could be organized into multiple protocols. Questionnaires were answered and tests were performed on a computer screen, both integrated via a special software with wearable sensors including ECG and EEG sensors that participants put on before the test (FIPOK system, V22-C, Aviatronic Kft, Budapest, Hungary). ECG sensors (five-channel Shimmer3 ECG Unit, Shimmer Research Ltd., The Realtime Building, Clonshaugh Business & Technology Park, Dublin, Ireland) [31] attached via a chest strap were used in the present study.

Answering questions or performing tasks on the screen could be stopped at any time and/or sensors could be removed. Four separate measurement units with the same pre-organized protocol coordinated by a central computer enabled the assessment of 4 persons at a time without disturbing each other. The measurement system was placed in a secure tempered room with four separate desks (work stations) for the four measurement units.

All participants received detailed information before testing. Participation in the study was anonymous and voluntary; participants were asked to create an ID from letters and numbers for themselves. The protocol of cognitive tests used in this research consisted of 11 tasks described in Section 2.4.2. Measurements were performed between March 2024 and September 2025. Individual measurements lasted approximately 60 min, not including the duration of informing the participants and placing/removing the wearable sensors.

### 2.4. Data Sources/Measurement

#### 2.4.1. Computer-Adapted Questionnaire

Data on the following items was collected by questionnaire: age, gender, year of birth, dominant hand (right or left), frequency of video game use (6 categories from never to daily), level of study (bachelor, master, undivided, doctoral), faculty of studies (13 faculties of the University contracted into 3 major fields of study), sleep duration (“How long did you sleep last night” (in hours), feeling rested (Do you feel rested?—not rested, rested, over-rested contracted into 2 categories: not rested, rested). Participants also filled the 12-item version of the General Health Questionnaire (GHQ-12) that measures psychological distress rated on a four-point Likert scale providing a total score ranging between 12 and 48 points [32].

#### 2.4.2. Protocol of Gamified Cognitive Tests

The protocol consisted of screen-adapted questionnaires, gamified versions of standard paper-based tests and 1 min relaxation at the beginning and end of testing. To specify the assessed functions, gamified tests were allocated to cognitive functions using the Research Domain Criteria (RDoC) matrix of human functions [33] and the Cattell–Horn–Carroll (CHC) theory of cognitive abilities [34] as follows:

1. Stroop (color–word inhibition) is the digital version of the original Stroop task [35] in which out of three ink colors of color words 2 are mismatched, and the participant must choose the matching word. The test measures cognitive control (performance monitoring subconstruct) according to the RDoC matrix, and visual processing (Gv), working memory (Gsm) and processing speed (Gs) of the CHC model [36].
2. Corsi block-tapping test is the digital form of the Corsi block-tapping test [37]. Participants must click on the blocks as the patterns are presented. This test measures working memory (active maintenance, limited capacity, interference control) according to the RDoC, and visual processing (Gv), working memory (Gsm) and processing speed (Gs) in the CHC model.
3. Character memory task is the gamified version of Digit Span Task [38] in which icons and numbers are presented at the same time after which participants must recall them in order. This task measures working memory in the RDoC matrix (Subconstructs: Active Maintenance, Flexible Updating, Limited Capacity, Interference Control); and working memory (Gsm) and processing speed (Gs) in the CHC model.
4. Grammatical Reasoning: Validity of logic statements need to be determined for each presented example. The original task of Baddeley was implemented [39]. According to the RDoC matrix, this task measures language abilities (comprehension, experimental manipulations), and reading–writing (Grw, requires reading fluency and passage comprehension) according to the CHC model [40].
5. Shape Memory Task: The participant is presented with three symbols and is expected to memorize them. Then the participant will select the proper combination out of four possibilities [41,42,43]. This task measures working memory in the RDoC matrix (Subconstructs: Active Maintenance, Flexible Updating, Limited Capacity, Interference Control); and working memory (Gsm) and processing speed (Gs) in the CHC model.
6. Trail making A (TMTA): The participant is presented with numbers from 1 to 24 located randomly on the screen. The participant is expected to click on the number in increasing order. This along with the trail making B test was developed to test the impact of brain damage [44]. The test assesses attention and cognitive control (subconstructs: Response Selection; Inhibition/Suppression) in RDoC, and processing speed (Gs) in the CHC model.
7. Trail making B (TMTB): The participant is presented with numbers from 1 to 12 and letters from A to L randomly located on the screen. The participant is expected to click on the numbers and letters in increasing order by starting with number 1 and then A, switching between numbers and letters [44]. This test assesses attention and cognitive control (subconstructs: Goal Selection; Updating, Representation, Inhibition/Suppression and Maintenance) according to RDoC; working memory (Gsm) and processing speed (Gs) according to the CHC model are measured by this task.
8. Numerical reasoning: This test is a simple version of the widely used numerical reasoning test that is usually part of various aptitude tests [45]. The participant is presented with single-digit computations where some of the numbers are given as text, others are written as digits. This task measures basic arithmetic operations to assess cognitive deficits [46] and language in RDoC; reading–writing (Grw, math fluency) [40] in CHC.
9. Monotony grid: This task is a slightly modified version of the Psychomotor Vigilance Test developed for assessing the impact of sleep loss on performance during sustained work [47]. The participant is presented with a grid of horizontal and vertical columns delineating cells. These cells light up one at a time in varying order, and the participant is expected to click on the lit-up cell. Attention and motor actions (reaction time) are assessed according to the RDoC; and processing speed (Gs) according to the CHC.
10. Candy counter: This is a visual scanning task [48] during which the participant sees several candies in different colors and patterns at random locations on the screen. Then the screen is obscured for a brief period after which in addition to the original set of candies, a new candy appears on the screen. The participant is expected to click on the newly added candy. Working memory (active maintenance; limited capacity) is assessed according to both the RDoC and the CHC.
11. Highway drive: This task is a video racing game used to assess risk-taking [49]. The participant can control a car—which travels along a three-lane highway—via the keyboard by changing the speed and lane of the car. In addition to the participant’s car, other vehicles are slowly moving in all lanes in the same direction. The participant is instructed to travel as far as possible during the allocated time while avoiding other cars as the participant’s car slows down in the case of a collision. Attention, cognitive control, and motor action are assessed according to the RDoC.

#### 2.4.3. ECG Data

ECG data were obtained by Movesense Medical, a Bluetooth-equipped single-channel ECG sensor [50] that was put on a wearable chest strap and worn during the test by each participant. The sensor provided clinical-grade ECG (sampling frequency: 128 Hz, RR intervals: 200–2000 ms) and heart rate (range: 20–240 bpm) data collected by the psychophysiological system. Heart rate variability metrics, specifically root mean square of the successive differences (RMSSD) [9] and Baevsky Stress Index (BSI) [51] were calculated and used in the statistical analysis.

### 2.5. Statistical Analysis

Demographic data were used as interval (e.g., age, sleep duration) or categorical (e.g., sex, level of study, feeling rested) variables. Cognitive test results were calculated for each participant as percent performance based on reference data from the literature (percent achieved of the maximum score).

Statistical analysis was conducted in Jamovi 2.4.11.0 (Sydney, Australia). We tested normality with the Kolmogorov–Smirnov test and chose parametric statistical test for normally distributed, and nonparametric test for non-normally distributed variables. To determine differences between two groups we used independent sample *t*-test (parametric) or Mann–Whitney test (nonparametric). Pearson and Spearman tests were performed for correlation analysis.

## 3. Results

### 3.1. Description of Participants

A total of 107 participants were included in the analysis. The mean age was 22.2 years (*SD* ± 2.22) ranging from 18 to 29 years. Participants’ university entrance score averaged 433 points (*SD* ± 36.98), with a minimum of 285 and a maximum of 494. The mean of sleep duration was 6.34 h (*SD* ± 1.27) ranging from 4 to 9.5 h (Table 1). Of the sample, 52% were females. Faculties were contracted into three major fields of study. Students of the Faculty of Medicine (*n* = 38), Faculty of Pharmacy (*n* = 4), Faculty of Dentistry (*n* = 3) were allocated to medical and health sciences (*n* = 45). Students of the Faculty of Informatics constituted a separate group (*n* = 32). Students of the Faculty of Humanities (*n* = 4), Faculty of Law (*n* = 1), Faculty of Science and Technology (*n* = 3) and Faculty of Music (*n* = 22) were collated into the third group of Humanities and Science (*n* = 30). Close to half of them (44%) studied at the bachelor level, and 37% were medical students (“undivided”). The dominant majority, 88% of the sample, was right-handed, and approximately one-third (35.5%) never played video games. Close to two-third of the students (60%) stated that they were not well-rested (Table 2).

### 3.2. Description of Cognitive Performance and Sleep Duration

All participants completed the protocol detailed in Methods apart from the monotony grid task for which data from four participants were not completed. Descriptive statistics for each task are expressed as mean percent performance as described in Methods. The highest mean performance was achieved on the trail making A test, and the lowest mean performance was obtained in the highway drive test as shown in Table 3.

Students who felt well-rested had significantly longer sleep duration than those who did not (Student’s *t*-test *p* < 0.001) as shown in Table 4.

### 3.3. Association Between Being Stressed, Feeling Rested, Sleep Duration and Cognitive Performance

The mean of the total GHQ-12 scores of students who felt rested were significantly lower (24.0, *SD* ± 6.43), that is, they were significantly less stressed compared to the scores of those who did not feel rested (26.8, *SD* ± 6.56; *p* = 0.032). Participants who reported being well-rested showed significantly higher overall cognitive performance compared to those who did not feel well-rested (Student’s *t*-test *p* = 0.024). Feeling rested significantly correlated with sleep duration (Spearman’s *rho* = 0.326, *p* < 0.001). However, sleep duration was not directly associated with cognitive performance (Pearson’s *r* = −0.046; *p* = 0.635).

### 3.4. Association Between Cognitive Performance, Heart Rate Variability, Sleep Duration and Restedness

Valid ECG data were obtained for 102 individuals from which parameters for heart rate variability (HRV) were calculated as described in Methods. Prior to testing, basal HRV metrics were recorded during a 1 min relaxation period. The mean heart rate was 83.6 beats per minute (bpm) (*SD* ± 12.79) ranging from 54.94 to 116.9 bpm, the mean Baevsky Stress Index was 11.4 (*SD* ± 4.43; range: 4.8–26.4), and the mean of RMSSD was 36 (*SD* ± 16.64; range: 8.16–91.3). There was no significant association between overall cognitive performance and resting HRV.

Mean heart rate during the entire assessment was 84.38 bpm (*SD* ± 12.06) ranging from 61.80 to 118.10 bpm. Heart rate showed no correlation with overall cognitive performance (*r* = 0.157, *p* = 0.115). The mean Baevsky Stress Index was 11.91 (*SD* ± 4.47; range = 5.19–24.40) (higher values of BSI reflect higher autonomic stress load). The BSI showed a significant positive association with overall cognitive performance (*r* = 0.195, *p* = 0.050). In contrast, the root mean square of the successive differences or RMSSD, a parasympathetic HRV marker with a mean of 33.09 ms (*SD* ± 15.19; range = 10.65–80.80) (higher values of which show lower autonomic stress load) was inversely associated with cognitive performance (*r* = −0.195, *p* = 0.050) (Table 5).

When repeating the correlation analysis after dropping two outliers of cognitive performance (highest: 81.8%, lowest: 36.1%), the strength of association between both cognitive performance and BSI (Pearson’s *r* = 0.218, *p* = 0.030) and cognitive performance and RMSSD (Pearson’s *r* = −0.211 *p* = 0.035) improved.

There was no difference in heart rate and HRV parameters (HR, BSI, RMSSD) between well-rested and not-rested groups; similarly, HRV parameters did not correlate with sleep duration. There was no significant association either between the total GHQ-12 score and HRV parameters (Baevsky index, *p* = 0.304; and RMSSD, *p* = 0.285) or between GHQ-12 and cognitive performance (*p* = 0.542).

## 4. Discussion

The present study explored the relationship between cognitive performance, sleep duration along with the subjective feeling of being rested, and heart rate variability as a measure of stress among university students using a novel multimodal measurement system. Instead of sleep duration, the feeling of rested correlated with higher cognitive performance suggesting that the perception of being rested may capture aspects of sleep quality that sleep duration does not. Lower autonomic stress load assessed by HRV parameters also showed positive correlation with better cognitive performance. These findings together suggest complex associations between autonomic and neural physiological markers and global cognitive functioning.

One strength of the study is the use of an innovative, multimodal system in which cognitive and psychophysiological measures were collected in a synchronized and integrated manner. A diverse battery of standard gamified cognitive tasks was used to produce comparable results in a relatively homogeneous sample of young healthy persons that reduced confounding by age and health status. However, limitations must also be addressed, such as the fact that sleep-related parameters were measured by self-report single-item questions. ECG was analyzed during task performance and not at rest; therefore, individual differences between baseline autonomic stress load could not be accounted for. Additional limitations are derived from the fact that the health status of the students was assumed to be good and free from major diseases impacting cognitive performance rather than investigated. The cross-sectional study design allows conclusions only on association but not causal inference between behavioral state (being rested), stress and cognitive performance. Precision regarding stress levels could be increased by measuring cortisol levels but this would require a different study design, more resources, and much greater commitment from participants [52].

Similarly to our results, single-night sleep duration before a test did not affect test performance among US college students but the pattern and quality of sleep in a month or week mattered the most [53].

June and colleagues compared high school students in an experimental group that underwent sleep restriction and a control group that did not. They found that sustained attention in the experimental group after two recovery nights did not return to the previous level, underlying the importance of having a regular healthy sleep habit [54]. The importance of subjective judgment of sleep quality in terms of tiredness or feeling restored on waking and during the day was described among insomnia patients and normal sleepers [55]. Sleep quality was found to be significantly correlated with the risks of anxiety and depression in a large sample of university students in the UK during four years of follow-up [56].

Another notable finding is that parameters of heart rate variability reflecting higher stress levels (lower RMSSD or higher Baevsky Stress Index) were associated with higher cognitive performance and may suggest a mild arousal-related enhancement of performance consistent with the classical Yerkes–Dodson law. In addition, this finding has been in alignment with the findings of others. A systematic review of 12 relevant papers published up to 2023 concluded that all examined studies found a longitudinal relationship between heart rate variability and cognition [57]. The association between cognitive load and stress levels assessed by HRV requires further studies. Increasing awareness about the importance of good quality sleep and stress reduction measures among college students could contribute to the uptake of non-invasive [58] rather than chemical [59] means of improving their cognitive performance.

## 5. Conclusions

The present study provides evidence on the relation between the perception of being rested, cognitive performance and objectively measured stress levels, also demonstrating the feasibility and utility of a novel integrated psychophysiological measurement system for evaluating cognitive performance along with psychophysiological functions among university students. The integration of gamified cognitive tasks with wearable sensors allowed for the successful simultaneous assessment of self-reported states, and objective data on cognitive performance and stress physiology. The subjective perception of “being rested” was a significant predictor of better cognitive performance. The impact of sleep on cognitive output is likely mediated by the perception of feeling rested rather than the duration of sleep.

## Figures and Tables

**Table 1:** Characteristics of participating students (interval variables).

Characteristics	*n* (%)	Mean	Min	Max
Age	107 (100%)	22.2 (*SD* ± 2.22)	18	29
Entrance score *	57 (53%)	433 (*SD* ± 36.98)	285	494
Sleep duration	107 (100%)	6.34 (*SD* ± 1.27)	4	9.5

* Obtained at the entrance examination before starting studies.

**Table 2:** Characteristics of participating students (categorical variables).

Characteristics	Subcategory	*n* (%)	Characteristics	Subcategory	*n* (%)
Sex	Female	56 (52%)	Dominant hand	Right	94 (88%)
Field of Study	Medical and health sciences	45 (42%)		Left	11 (10%)
	Informatics	32 (30%)		Ambidextrous	2 (2%)
	Humanities and science	30 (28%)	Frequency of playing video games	Daily	15 (14%)
Study level	Bachelor	47 (44%)		Weekly	2 (1.9%)
	Master	12 (11%)		Often	15 (14%)
	Undivided	40 (37%)		Monthly	11 (10.3%)
	Doctoral	3 (3%)		Rarely	26 (24.3%)
	Other	2 (2%)		Never	38 (35.5%)
			Feeling rested	Not well-rested	64 (60%)
				Well-rested	43 (40%)

**Table 3:** Cognitive performance of the participants.

Tasks	*n* (%)	Mean% ± *SD*	Median	Min	Max
1. Stroop	107 (100%)	82.5 ± 4.42	83	72	90
2. Corsi	107 (100%)	81.2 ± 8.42	81	61	100
3. Character memory	107 (100%)	52.1 ± 11.33	52	27	85
4. Grammatical reasoning	107 (100%)	57.4 ± 30.79	60	0	100
5. Shape memory	107 (100%)	51.9 ± 9.24	54	7	67
6. TMT-A	107 (100%)	83.1 ± 17.25	90	25	100
7. TMT-B	107 (100%)	64.9 ± 26.57	70	5	100
8. Numerical reasoning	107 (100%)	65.4 ± 16.46	70	10	100
9. Monotony grid	103 (96%)	33.7 ± 19.08	29	7	82
10. Candy counter	107 (100%)	52.8 ± 14.94	50	21	86
11. Highway drive	107 (100%)	32.4 ± 8.58	34	9	50
Overall cognitive performance	107 (100%)	57.6 ± 8.70	58.9	36.1	81.8

**Table 4:** Sleep duration in hours and feeling rested by participants.

Sleep Duration	*n* (%)	Mean ± *SD*	Median	Min	Max
Of those who were not well-rested	64 (60%)	5.98 ± 1.17	6	4	9
Of those who were well-rested	43 (40%)	6.86 ± 1.25	7	4.5	9.5
Total	107 (100%)	6.34 ± 1.27	6.5	4	9.5

**Table 5:** Description of parameters of heart rate variability (HRV) and correlation with overall cognitive performance.

Parameter	*n* (%)	Mean ± *SD*	Median	Min	Max	Correlation (Pearson Test)
HRV parameters before testing (1 min relaxation)						
Heart Rate, HR (bpm)	102	83.6 ± 12.79	84.8	54.94	116.9	*r* = 0.116 *p* = 0.247
Baevsky Stress Index, BSI	102	11.4 ± 4.43	10.2	4.8	26.4	*r* = 0.165 *p* = 0.097
RMSSD	102	36 ± 16.64	33.3	8.16	91.3	*r* = −0.119 *p* = 0.234
HRV parameters during testing						
Heart Rate, HR (bpm)	102 (%)	84.38 ± 12.06	85.59	61.80	118.1	*r* = 0.157 *p* = 0.115
Baevsky Stress Index, BSI	102 (%)	11.91 ± 4.47	11.36	5.19	24.4	*r* = 0.195 *p* = 0.050
RMSSD	102 (%)	33.09 ± 15.18	30.55	10.65	80.8	*r* = −0.195 *p* = 0.050

## Data Availability

The datasets presented in this article are not readily available because the data are part of an ongoing study. Requests to access the datasets should be directed to the corresponding author.

## Abbreviations

The following abbreviations are used in this manuscript:

BSI	Baevsky Stress Index
HRV	Heart rate variability
HR	Heart rate (bpm)
RMSSD	Root Mean Square of the Successive Differences
